# Oligoclonal M bands and cervical spinal cord lesions predict early secondary progressive multiple sclerosis

**DOI:** 10.3389/fneur.2022.991596

**Published:** 2022-10-28

**Authors:** Carmen Alcalá Vicente, Laura Lacruz, Francisco Gascón, Sara Carratalà, Carlos Quintanilla-Bordás, Maria T. Sanz, María Carcelén-Gadea, Javier Mallada, Joan Carreres, Laura Gabaldón Torres, Jose Andres Dominguez, Emmanuel Cañizares, Sara Gil-Perotin, Laura Cubas, Raquel Gasqué Rubio, Jéssica Castillo-Villalba, Francisco Carlos Pérez-Miralles, Bonaventura Casanova

**Affiliations:** ^1^Neuroimmunology Unit, Polytechnic and University Hospital La Fe, Valencia, Spain; ^2^Neurology Service, Clinic University Hospital of València, Valencia, Spain; ^3^Didactics of Mathematics Department, University of València, Valencia, Spain; ^4^Neurology Service, General Hospital of València, Valencia, Spain; ^5^Neurology Service, General Hospital of Elda, Alicante, Spain; ^6^Radiology Service, Polytechnic and University Hospital La Fe, Valencia, Spain; ^7^Neurology Service, San Francesc de Borja Hospital, Valencia, Spain; ^8^Neurology Service, La Ribera Hospital, Valencia, Spain

**Keywords:** OCMB, oligoclonal M bands, spinal cord, secondary progressive MS, multiple sclerosis

## Abstract

**Objective:**

To determine baseline cerebrospinal fluid and magnetic resonance imaging (MRI) variables at the onset of a clinically isolated syndrome (CIS) suggestive of multiple sclerosis (MS) that predict evolution to secondary progressive MS (SPMS).

**Methods:**

276 CIS patients with a minimum follow-up of 10 years were studied. Baseline presence of oligoclonal IgG and IgM bands (OCGB and OCMB respectively); number of brain T2 lesions (B-T2L), brain gadolinium enhancement lesions (brain-GEL), cervical spinal cord T2 lesions (cSC-T2L); and fulfillment of 2017 McDonald criteria among other variables were collected.

**Results:**

14 patients ended up with a non-MS condition. 138/276 CIS patients fulfilled 2017 McDonald criteria. Mean age was 32.4 years, 185 female. 227 received treatment, 95 as CIS. After a mean follow-up of 12 years, 36 patients developed SPMS. Conversion to SPMS was associated with OCGB (p = 0.02), OCMB (p = 0.0001); ≥ 9 B-T2L (p = 0.03), brain-GEL (p = 0.03), and cSC-T2L (p = 0.03). However, after adjusting for sex, age, BT2L, brain-GEL, SC-T2, and OCMB status, only OCMB (HR 4.4, 1.9–10.6) and cSC-T2L (HR 2.2, 1.0–6.2) suggested an independent association with risk of conversion to SPMS. Patients with both risk factors had a HR of 6.12 (2.8–12.9).

**Discussion:**

OCMB and SC-T2 lesions are potential independent predictors of conversion to SPMS.

## Introduction

Transition to secondary progressive multiple sclerosis (SPMS) is one of the worst-case scenarios a patient with relapsing-remitting multiple sclerosis (RRMS) can encounter, as disease-modifying therapies (DMTs) have shown very limited efficacy to reduce the progression of disability during this phase. Avoiding transition to SPMS is critical to stop disability accrual in the long term. Unfortunately, most DMTs have proven futile to reduce this risk. In this regard, some studies suggest that early treatment with highly effective DMTs (he-DMTs) dramatically decreases the proportion of patients evolving to SPMS, although these drugs are also associated with serious adverse events (1, 2). This prompts the identification of patients at risk of SPMS at disease onset to design a therapeutic strategy with a balanced risk-benefit (3). This study aims to identify early magnetic resonance imaging (MRI) and cerebrospinal fluid (CSF) predictors of conversion to SPMS.

## Methods

### Design

This is a multicentric study from six multiple sclerosis (MS) units in the Valencian Land, Spain. All patients who presented with an episode suggestive of clinically isolated syndrome (CIS) between 2000 and 2005 were identified retrospectively. Follow-up data until study entry were obtained retrospectively and then, prospectively, for a minimum follow-up of 10 years.

The CIS patients were selected regardless of fulfillment of the 2017 McDonald criteria (4). Patients without baseline CSF, brain and cervical spinal cord (SC) MRI studies before the second relapse, and treatment initiation were excluded. All patients underwent extensive workup to exclude alternative diagnosis, including cell-based assay for anti-AQP4 antibodies when neuromyelitis optica was suspected.

The CSF study included the determination of oligoclonal IgG bands (OCGB), oligoclonal IgM bands (OCMB), and lipid-specific oligoclonal M bands (LS-OCMB) in CSF. MRI variables included baseline number of brain T2 lesions (B-T2L), brainstem lesions, brain gadolinium-enhancing lesions (brain-GEL), and T2 cervical spinal cord lesions (cSC-T2L).

Patients were followed up at every 3 months visits and whenever the patient considered necessary because of worsening of their neurological symptoms. Treatment was selected according to the approved indications of the Health Ministry of the Valencian Country. In general, first-line therapy was offered to all cases of first relapse (CIS) with a high risk of conversion to relapsing-remitting multiple sclerosis (RRMS), regardless of fulfilling their contemporary diagnostic criteria. High risk of RRMS was defined as having more than 9 lesions in B-T2L and the presence of OCGB. He-DMT was initiated in cases of treatment failure, defined as either having a relapse and brain-GEL in an MRI performed at least 3 months apart or having more than one relapse within a year.

A relapse was defined by the presence of new symptoms or worsening of previous neurological symptoms lasting more than 24 h in the absence of fever, or any other condition that could explain the worsening.

The SPMS diagnosis was defined as an increase in the Expanded Disability Status Scale (EDSS) of 1.0 points, confirmed at 3 months, not due to a relapse, with a pyramidal system higher or equal to 2.0 and a minimum EDSS of 3.0 (5). The Multiple Sclerosis Severity Score (MSSS) and annualized relapse rate (ARR) were also calculated.

The following clinical and demographic variables were explored: age at presentation, sex, topography of CIS (myelitis, optic neuritis, brainstem syndrome, polyregional, or indeterminate), and timing of DMT initiation (before vs. after the second relapse). EDSS values were recorded at year 5, year 10, and at the last follow-up visit.

Age at presentation was grouped into < 30, 30–39, and >39 years, in accordance with the natural history studies that show the first attack usually occurs around 30 years and the clinically evident progression occurs around 40 years (6).

The MRI variables analyzed were the number of B-T2L, the presence of infratentorial lesions, brain-GEL, and the number of cSC-T2L. CSF variables were the presence of OCGB, OCMB, and LS-OCMB.

The B-T2L were stratified into 1, 2–9, and >9 lesions, in accordance with the studies that show that having 10 or more lesions at baseline is associated with poor prognosis in both the short term and long term (7).

### MRI studies

The analyzed studies were acquired over 10 years using two models of magnetic resonance scanners (GE Signa HDxt 1.5 tesla). Brain imaging protocols included 3D FLAIR sagittal (slice thickness 1 mm) and post-gadolinium axial T1 sequences (slice thickness 3 mm) with a concentration of 0.2 mmol/kg. The cervical spinal cord was analyzed with axial and sagittal T2 sequences (3 mm slice thickness, both).

An experienced blinded neuroradiologist in MS reviewed and reported the number of lesions. In addition, two blinded neurologists also reviewed the images.

### Cerebrospinal fluid determinations

Paired samples of serum and CSF were aliquoted and stored at −80°C until the assay was performed. OCMB were detected by isoelectric focusing (IEF) and immunodetection according to the technique described by Villar et al. (8). Briefly, serum samples were diluted in saline before the IEF in order to reach the same concentration range as in CSF samples. Focusing was performed on a Multiphor II Electrophoresis System (GE Healthcare, Chicago, USA) at pH 5 to 8. Then, proteins were transferred to a PVDF membrane by Western blot. Finally, immunodetection was performed by biotin-conjugated-goat anti-human IgM (Sigma) and streptavidin-alkaline phosphatase (Jackson ImmunoResearch).

### Study endpoints and statistical analysis

The main endpoint of our study was to determine which CSF and MRI parameters at the time of presentation of a CIS predicted conversion to SPMS. A multivariate model was constructed. To explore the influence of the variables in the model and to avoid internal correlation, we studied the influence of each variable with a chi-square analysis (Supplementary Table 1). Nine different models and their corresponding log-likelihoods were obtained. The highest log-likelihood values were obtained in models 3 (312.471) and 4 (331.111). However, the beta-coefficient was only significant in model 3 (Supplementary Table 2) and thus was selected. This model comprised the following variables: fulfillment of the 2017 McDonald criteria at presentation, B-T2L, cSC-T2L, brain-GEL, OCGM, and OCMB. Because OCGB were present in more than 84% of patients, we considered that they were not discriminative and were substituted for age. To validate this decision, we performed an omnibus test of the two models. This showed that the model that included age at onset was more informative than the presence of OCGB (log-likelihood 276,968 vs. 275.847, respectively; Supplementary Tables 3, 4). Since the presence of LS-OCMB did not provide any additional value compared to OCMB, we excluded this variable from the analysis (Supplementary Table 5).

After obtaining the most informative variables, we performed the Kaplan-Meier survival analysis to calculate the time to reach SPMS. We first calculated the variables at baseline that predicted the evolution to SPMS. To do this, we performed the Student's *t*-test and chi-square test to explore the influence of the variables. Then, we conducted univariate and multivariate (adjusted for sex and age). Cox regression analyses to explore the putative clinical value of MRI and CSF baseline predictor variables. The absolute and relative risks were also calculated.

Type of treatment was also analyzed. It was stratified according to the efficacy of initial treatment (“first-line DMT” and he-DMT). Beta-interferon and glatiramer acetate were considered the first-line DMTs, while natalizumab and fingolimod were considered he-DMTs. Rituximab and ocrelizumab, which became available during follow-up, were also considered he-DMT.

In addition to baseline predictors, we also analyzed the contribution of evolution-related predictors such as the ARR and EDSS at year 5, year 10, and at the end of the follow-up to the risk of conversion to SPMS.

### Data availability

All document data, methods, and materials used to conduct the research in this article are available in the main article or in the Supplementary material.

## Results

### Patient's characteristics

**Figure 1:**
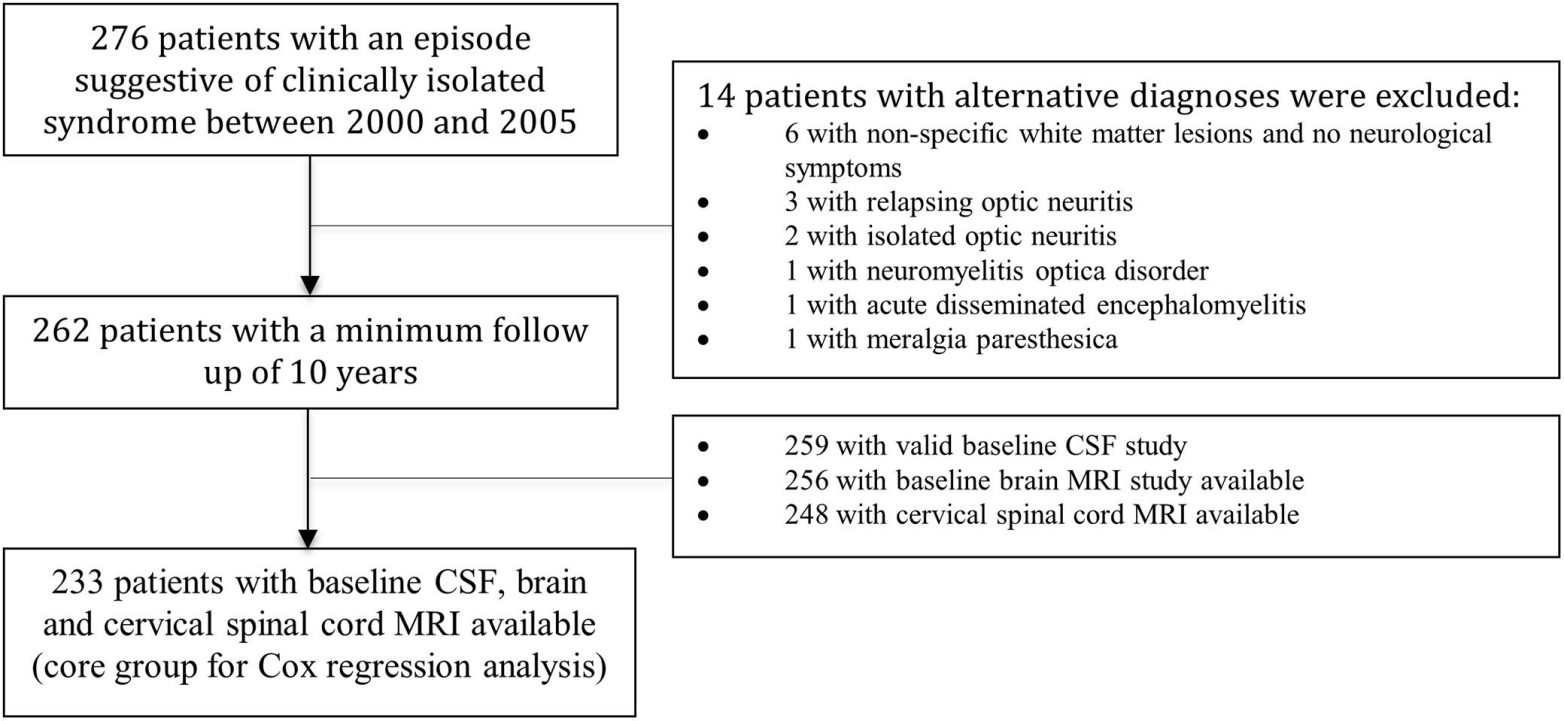
Flowchart of the patients included in the study.

In total, 276 patients with CIS between 2000 and 2005 were identified. During follow-up, 14 patients were diagnosed with non-MS conditions and therefore were excluded. The remaining 262 patients were included in the analysis and had a mean follow-up of 12.8 (SD 2.9) years; 259 patients had baseline OCB study in CSF, 256 had baseline brain MRI, and 248 had SC MRI; 233 (88.9%) patients had all three studies available (CSF, brain, and SC MRI) and conformed the core group for the multivariate Cox analysis. A summary of the flowchart of patients is shown in Figure 1. Clinical and demographic characteristics of the cohort are summarized in Table 1.

**Table 1:** Clinical and demographic characteristics of the patients studied.

	**Total, *n* = 262**	**Remained as RRMS or CIS, *n* = 226**	**Reached SPMS diagnosis, *n* = 36**	***p*-value**
**Baseline demographic characteristics** ***n*** **=** **262**				
Age^*^	32.4 (9.1)	32.1 (9.1)	33.7 (9.2)	ns
Sex (females)	185 (70.6)	158 (59.9)	27 (75.0)	ns
Evolution time (years)^*^	12.8 (2.9)	12.7 (2.8)	13.2 (3.1)	ns
Clinical characteristics				
Topography of CIS, *n* = 249				ns
Myelitis	91 (36.5)	77 (35.3)	14 (45.2)	
Optic neuritis	47 (18.9)	45 (20.7)	2 (6.3)	
Brainstem syndrome	67 (26.9)	57 (26.3)	10 (31.3)	
Polyregional syndrome	21 (8.4)	18 (8.3)	3 (9.4)	
Hemispheric syndrome	23 (9.2)	21 (9.7)	2 (6.3)	
ARR, mean (SD)	*0.25 (3.9)*	*0.12 (4.9)*	*1.0 (2.2)*	0.03^**^
Median (IQ)	*0.27 (0.1–0.5)*	*0.25 (0.4–0.5)*	*0.58 (0.2–1.1)*	
Baseline EDSS	*1.5 (0.9)*	*1.4 (0.8)*	*2.4 (0.9)*	* < 0.000*
EDSS at year 5	*1.9 (1.1)*	*1.8 (1.0)*	*2.7 (1.2)*	*0.0001*
EDSS at year 10	*1.9 (1.4)*	*1.6 /(1.1)*	*3.7 (1.6)*	* < 0.0000*
EDSS at last observation	*2.2 (1.6)*	*1.7 (1.2)*	*4.9 (1.5)*	* < 0.0000*
MSSS	*3.0 (2.3)*	*2.6 (2.0)*	*5.7 (2.5)*	* < 0.000*
**Baseline MRI characteristics**, ***n*** **=** **256**				
Time between CIS and MRI, median –(IQ), days	92 (14.5–280.0)	68 (12.5–254)	145.5 (23.7–437)	Ns
Total number of B-T2L^*^	13.0 (11.7)	11.7 (9.7)	21.0 (18.5)	0.006
NT2L (grouped)				0.005
0–1 B-T2L	26 (10.2)	26 (11.8)	0 (0.0)	
Between 2 and 9 B-T2L	87 (34.0)	81 (36.8)	6 (16.7)	
> 9 B-T2L	143 (55.9)	114 (51.8)	29 (80.6)	
Scans with brain GEL	143 (56.3)	116 (53.0)	27 (77.1)	0.007
Scans with BS-T2L	116 (47.2)	97 (46.2)	19 (52.8)	ns
Scans with cSC-T2L (*n* = 248)	145 (58.5)	117 (55.2)	28 (77.8)	0.011
Number of cSC-T2L^*^	1.1 (2.2)	1.03 (1.3)	1.75 (1.5)	0.012
**Baseline cerebrospinal fluid characteristics**				
Presence of OCGB (*n* = 259)	219 (84.6)	185 (86.5)	34 (97.1)	0.027
Presence of OCMB (*n* = 253)	124 (49.0)	99 (45.0)	25 (75.8)	0.001
Presence of LS-OCMB (*n* = 243)	91 (37.4)	73 (34.4)	18 (58.1)	0.016
Fulfillment of McDonald 2017 criteria at baseline (*n*, %)	138 (53.9)	111 (50.2)	27 (77.1)	0.003
**Treatment**, ***n*** **=** **262**				
Received treatment	227 (86.6)	191 (84.9)	36 (97.3)	0.040
Initiated treatment as CIS^+^	95 (36.2)	79 (41.4)	16 (44.4)	ns
Initiated treatment with he-DMT ^Δ^	15 (5.7)			
**Evolution**, ***n*** **=** **262**				
Treatment at last follow up				
No treatment	35 (13.4)	35 (15.5)	0 (0.0)	
1sr line therapies	125 (47.7)	114 (50.4)	11 (30.6)	
He-DMT^Δ^	102 (38.9)	77 (34.1)	25 (69.4)	
Phenotype at last follow up				
CIS^+^	21 (8.0)			
Fulfilled 2017 McDonald criteria	205 (78.2)			
SPMS	36 (13.7)			

Values expressed in brackets represent percentages unless stated otherwise.

* Mean and standard deviation.

** Mann-Whitney U-test.

+ Not fulfilling the 2017 McDonald criteria.

Δ High efficacy disease-modifying treatments that include fingolimod, natalizumab, rituximab, and ocrelizumab.

B-T2L, brain T2 lesions; NT2L, number of brain T2 lesions; GEL, gadolinium-enhancing lesions in the brain. BS-T2L, brainstem and cerebellar T2 lesions; cSC-T2L, cervical spinal-cord T2 lesions; OCGB, oligoclonal G bands; OCMB, oligoclonal M bands; LS-OCMB, lipid-specific oligoclonal M bands; CIS, clinically isolated syndrome; RRMS, relapsing-remitting multiple sclerosis according to McDonald 2017 criteria; SPMS, secondary progressive multiple sclerosis; MSSS, Multiple Sclerosis Severity Scale; ns, nonsignificant.

All patients were Caucasian except for one that was Romani. At baseline, the mean age was 32.4 years (SD 9.1), and 185 (70.6%) patients were female; 138 (53.9%) patients fulfilled the 2017 McDonald criteria at presentation (retrospectively applied), 219 (84.6%) patients presented OCGB, and 124 (49%) patients presented OCMB; 91 (73.4%) patients with OCMB also had LS-OCMB. Only one patient had positive OCMB but negative OCGB and otherwise fulfilled the criteria for RRMS. A total of 227 (86.6%) patients initiated the treatment, of which 95 (36.2%) patients as CIS not fulfilling the 2017 McDonald criteria. Only 15 (5.7%) patients started treatment with he-DMT.

At the end of the follow-up, 241 (91.9%) patients fulfilled the 2017 McDonald criteria: 205 (78.2%) as RRMS and 36 (13.7%) as SPMS. Of the 42 patients with neither OCGB nor OCMB, only one converted to SPMS; 25 (69.4%) patients treated with he-DMT reached a diagnosis of SPMS, compared to 77 (34.1%) of those that remained as CIS/RRMS. Most CIS/RRMS patients (*n* = 38, 52.7%) were treated with fingolimod, while most SPMS patients were treated with an anti-CD-20 monoclonal antibodies [*n* = 14 (58%): 11 with rituximab and 3 with ocrelizumab].

The number of scans with baseline presence of cSC-T2L was 145 (58.5%). Among these, there were no demographic differences between patients that remained either as CIS, RRMS or converted to SPMS. Patients reaching SPMS diagnosis had at baseline more frequent fulfillment of the 2017 McDonald criteria (*p* = 0,003), higher number of B-T2L (21.0 vs. 11.7, *p* = 0.006), presence of brain-GEL (77.1% vs. 53.0%, *p* = 0.007), SC lesions (77.8% vs. 55.2%, *p* = 0.012), and OCGB and OCMB (97.1% vs. 86.5%, *p* = 0.027; and 75.8% vs. 45.0%, *p* = 0.001; respectively). However, no differences in time to reach SPMS were seen when comparing patients who started treatment when fulfilling the 2017 McDonald criteria or not.

MSSS and EDSS at year 5, year 10, and at the last observation were higher in patients who converted to SPMS. In RRMS patients, EDSS remained stable during the 12 years of follow-up (Table 1). Although baseline EDSS was higher in patients that evolved to SPMS with respect to those that did not (2.4 vs. 1.4, respectively), the introduction of this variable in the multivariate model did not change the predictive value of OCMB and T2-SCL (Supplementary Table 6). Patients who evolved to SPMS also had both higher absolute number of relapses [mean 3.0 (SD 1.3) vs. 2.5 (SD 1.3), *p* = 0.03 (Mann-Whitney *U*-test)] and ARR [mean 0.47 (SD 0.4) vs. 0.24 (SD 0.1), *p* = 0.00004] than those who remained as CIS/RRMS (Table 1).

**Figure 2:**
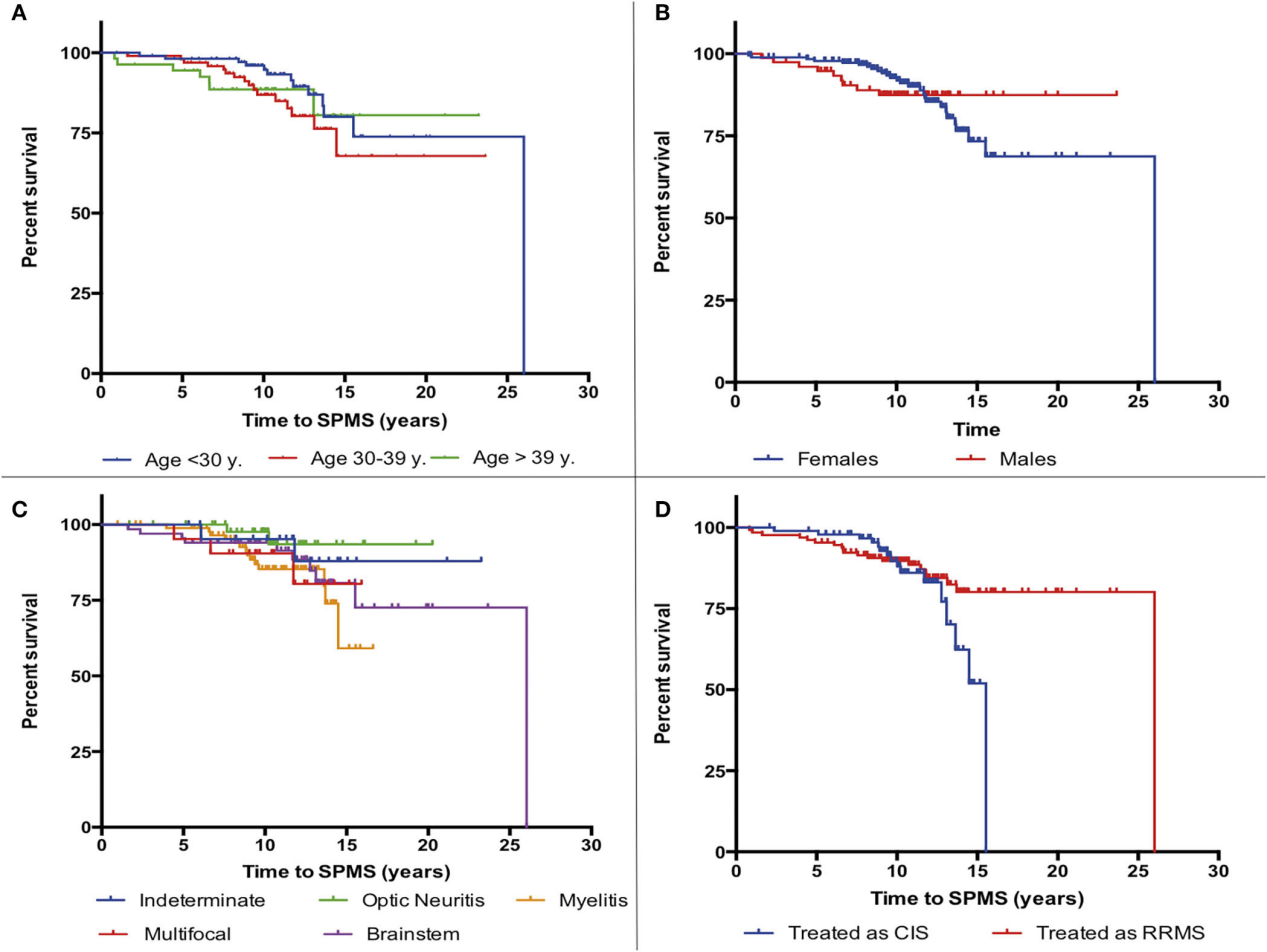
Kaplan-Meier survival analysis of the time to reach a diagnosis of secondary progressive multiple sclerosis (SPMS) according to demographic and clinical characteristics. **(A)** Age at first symptom, **(B)** sex, **(C)** initial clinical syndrome, and **(D)** be treated as clinically isolated syndrome (CIS) or as relapsing-remitting multiple sclerosis (RRMS).

**Figure 3:**
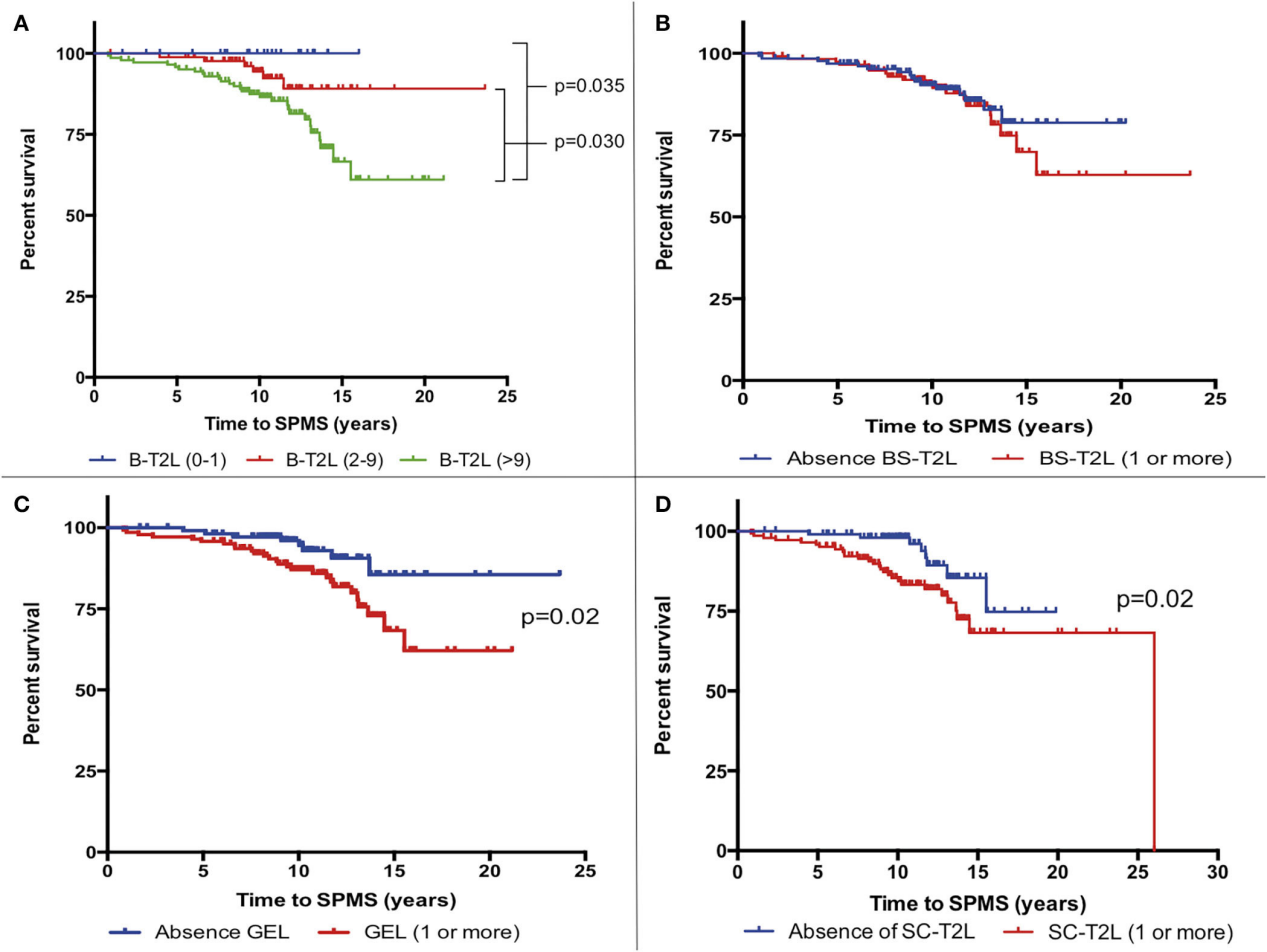
Kaplan-Meier survival analysis of the time to reach a diagnosis of secondary progressive multiple sclerosis (SPMS) according to MRI characteristics. **(A)** Number of brain T2 lesions (B-T2L). **(B)** Presence vs. absence of infratentorial lesions (BS-T2L). **(C)** Presence or absence of brain gadolinium-enhancing lesions (GEL). **(D)** Presence or absence of cervical spinal cord T2 lesions (cSC-T2L).

**Figure 4:**
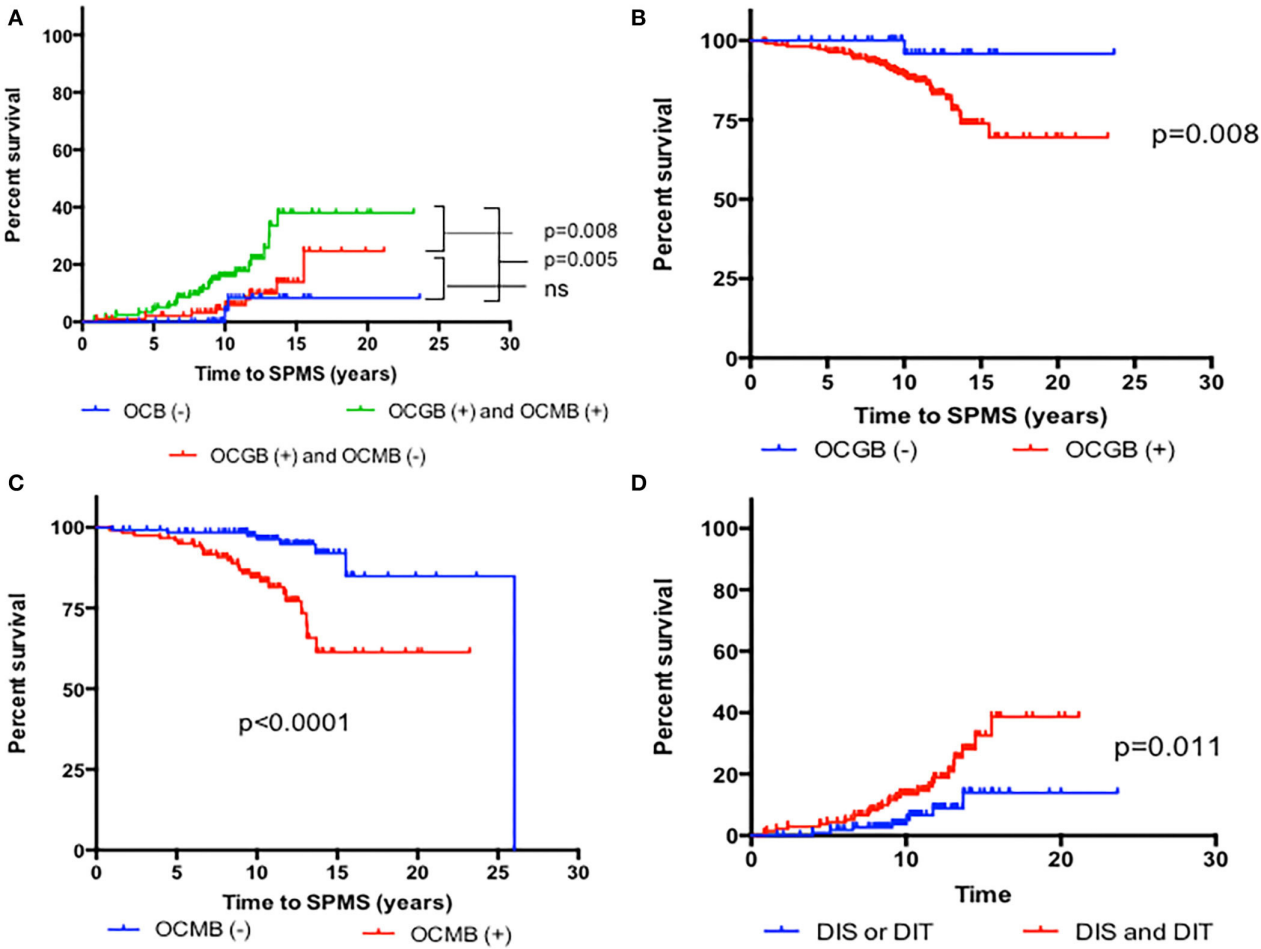
Kaplan-Meier survival analysis of the time to reach a diagnosis of SPMS according to cerebrospinal fluid characteristics. **(A)** Presence of both oligoclonal M bands (OCMB) and oligoclonal G bands (OCGB) vs. presence of only OCGB vs. absence of OCMB and OCGB. **(B)** Presence vs. absence of OCGB. **(C)** Presence vs. absence of OCMB. **(D)** Presence of dissemination in space (DIS) or time (DIT) alone, vs. presence or both according to 2017 McDonald at presentation.

The mean time among the 36 (13.9%) patients who reached a diagnosis of SPMS was 9.1 years (SD 4.8). The median survival time to reach SPMS according to the Kaplan-Meier estimator was 21.9 years. Presentation as optic neuritis was protective (*p* = 0.04), while the fulfillment of the 2017 McDonald criteria at presentation, or the presence of any of the following: more than 9 B-T2L or brain-GEL in the basal MRI, cSC-T2L, OCGB, and OCMB, was associated with earlier and more frequent conversion to SPMS (Figures 2–4).

**Figure 5:**
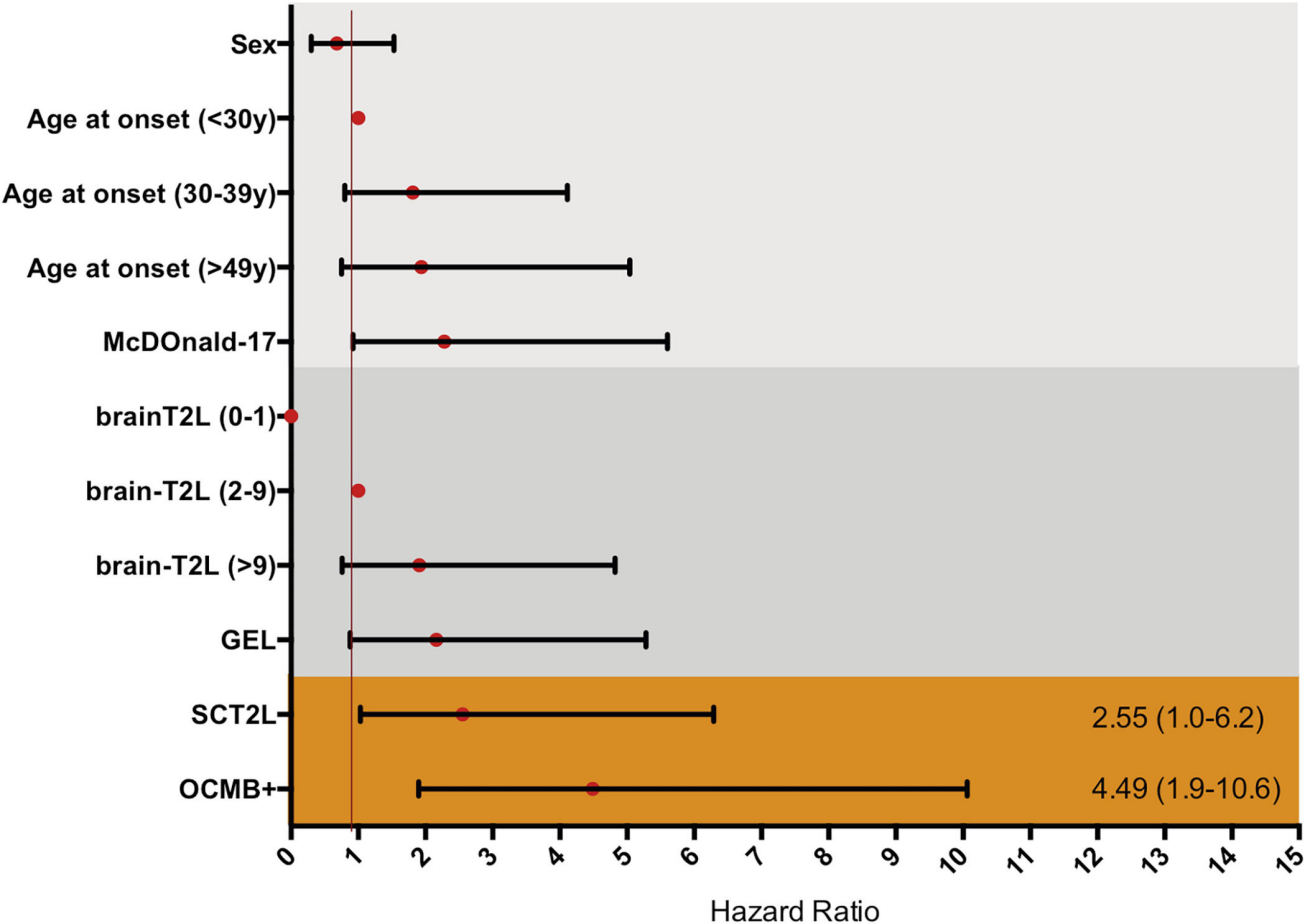
Multnivariate Cox proportional hazard ratio adjusted by sex and age, for each of the possible informative variables. McDonald-17, fulfillment of McDonald 2017 criteria at presentation; BrainT2L, brain T2 lesion; GEL, gadolinium enhancing lesion; SCT2L, presence of cervical spinal cord T2 lesion; OCMB+, presence of oligoclonal M bands.

A univariate Cox regression analysis was performed with the main variables (Table 2). Age, sex, and initial treatment were adjusted in multivariate Cox regression models to explore predictive variables. Fulfillment of the 2017 McDonald criteria, having more than 9 B-T2L, brain-GEL, cSC-T2L, or positive OCMB increased the risk of conversion to SPMS (Supplementary Figure 1). However, in the multivariate modeling using Cox regression analysis, after adjustment for age, sex, fulfillment of the 2017 McDonald criteria, B-T2L, brain-GEL, cSC-T2L and OCMB, only the presence of OCMB and cSC-T2L suggested independently a risk for the conversion to SPMS, with an HR of 4.4 (CI 1.9–10.6) for the presence of OCMB (*p* = 0.001) and an HR of 2.55 (CI 1.0–6.2) for the presence of cSC-T2L (*p* = 0.027; Table 2 and Figure 5).

**Table 2:** Univariate Cox regression analysis of reaching a diagnosis of secondary progressive multiple sclerosis diagnosis according to the main clinical variables.

	**Number**	**Events (%)**	**Mean (SD)**	**95% CI**	***p*-value**
Female (*n*, %)	185	27 (14.6)	21.5 (0.8)	19.8–23.2	ns
**Age at onset of CIS (years)**					ns
< 30	108	13 (12.3)	22.4 (1.0)	20.3–24.4	
30–39	99	16 (16.1)	19.5 (1.0)	18.0–21.5	
>39	55	7 (12.7)	20.2 (1.1)	20.4–22.4	
**CIS syndrome**					
Myelitis	91	14 (15.3)	14.7 (0.4)	13.7–15.6	
Brainstem	67	10 (14.9)	21.9 (1.3)	18.3–24.6	
Optic neuritis	47	2 (4.2)	19.5 (0.4)	18.5–30.5	0.04
Multifocal	21	3 (14.2)	14.5 (0.7)	13.0–15.9	
Indeterminate	23	2 (8.6)	21.5 (1.1)	19.4–23.7	
**Brain MRI**					
0–1 B-T2L	26	0	16.0 (0.0)	16.0–16.0	
Between 2 and 9 B-T2L	87	6 (10.7)	22.0 (0.6)	29.8–23.3	0.035
>9 B-T2l	143	29 (20.3)	17.2 (0.6)	15.9–18.5	0.030
GEL absent	111	9 88.19	29.6 (1.1)	18.3–22.9	
GEL present	143	27 (19.9)	17.3 (0.6)	16.0–18.6	0.039
BS-T2L absent	130	17 (13.1)	17.5 (0.6)	16.2–18.8	
BS-T2L present	116	19 (16.4)	19.2 (0.9)	17.3–21.1	ns
**Cervical spinal cord MRI**					
cSC-T2L absent	103	9 (8.7)	17.6 (0.7)	16.2–19.0	
cSC-T2L present	145	28 (19.3)	20.9 (0.9)	19.0–22.7	0.02
**Cerebrospinal fluid features**					
OCGB absent	40	1 (2.5)	22.6 (3.8)	15.1–30.2	
OCGB present	219	34 (15.5)	19.4 (0.6)	18.1–20.6	0.008
OCMB absent	129	9 (7.0)	22.7 (1.4)	19.9–25.6	
OCMB present	124	25 (20.2)	18.1 (0.9)	16.3–20.0	0.0001
**Treatment**					
Treatment as CIS	95	16 (16.8)	13.8 (0.3)	13.0–14.5	
Treatment as RRMS	132	20 (15.4)	22–5 (0.7)	21.0–24.0	ns
**Fulfillment of 2017 McDonald criteria at presentation**					
No	118	9 (7.6)	20.7 (1.1)	18.4–23.0	
Yes	138	27 (19.6)	17.2 (0.6)	15.9–18.6	0.020

B-T2L, brain T2 lesions; CIS, clinically isolated syndrome; GEL, gadolinium-enhanced lesions in the brain. BS-T2L, brainstem and cerebellar T2 lesions; cSC-T2L, cervical spinal-cord T2 lesions; OCGB, oligoclonal G bands; OCMB, oligoclonal M bands; LS-OCMB, lipid-specific oligoclonal M bands; RRMS, relapsing-remitting multiple sclerosis.

In our cohort, OCMB-positive patients had an absolute risk to develop SPMS of 23%; 25 of the 124 (20.0%) patients with positive OCMB were converted to SPMS, compared to 8 of the 129 (6.2%) patients without them. This resulted in a relative risk of 3.25 (95% CI: 1.55–6.83), or a 3-fold risk of converting to SPMS when OCMB were present. These patients also had higher number of cSC-T2L at baseline [1.3 (1.3) vs. 0.9 (1.4); *p* = 0.007].

With respect to the presence of cSC-T2L, 28 of the 145 (19.3%) patients with cSC-T2L developed SPMS, compared to 8 of the 103 patients (7.7%) not having them. This resulted in a relative risk of 2.49 (95% CI: 1.7–5.31) or a 2-fold risk of converting to SPMS. Furthermore, patients with both OCMB and cSC-T2L had an HR of 6.12 (95% CI: 2.8–12.9), higher than either of the risk factors alone [HR of positive OCMB: 4.77 (95% CI: 2.0–11.0), HR of SCT2L: 2.77 (95% CI: 1.2–5.1)].

We further investigated the effect of starting with he-DMT on the risk of conversion to SPMS. Only 15 (5.7%) patients started with he-DMT, which limits the interpretation of results. Still, treatment with he-DMT was associated with a reduced risk of conversion to SPMS compared to the first-line DMT or no treatment (Supplementary Table 7). After adjusting for SCT2, OCMB, and efficacy of initial treatment (he-DMT vs. first-line DMT) in a Cox-regression, he-DMT was still associated with a reduced risk of conversion to SPMS (Supplementary Table 8).

## Discussion

We studied several CSF and MRI-related variables at baseline in a cohort presenting with a CIS, followed up for more than 10 years. We found that only the presence of OCMB and cSC-T2L potentially predicted a higher risk of developing SPMS, with an HR of 2.5 and 4.4, respectively. Strikingly, the presence of both factors increased by 6-fold the risk of developing SPMS.

Our findings are consistent with two recent studies that demonstrated the value of cSC-T2L and the presence of OCMB as prognostic factors for the development of SPMS (3, 9). In the former, 162 patients were followed up for 15 years and showed that the presence of cSC-T2L was the most robust predictor of developing SPMS, together with the presence of brain-GEL. In the second study, 196 patients with a median follow-up of 12.6 years showed that intrathecal synthesis of IgM increased the likelihood by 2-fold of developing SPMS.

The study of cervical spinal cord lesions and OCMB in CSF conveys two different approaches to evaluate the risk of developing SPMS. The presence of cervical spinal cord lesions may bias patient selection toward a worse EDSS, which might favor an earlier diagnosis of SPMS, as motor pathways and sphincter control (both of which are highly represented by the EDSS) are usually affected. Indeed, SC injury may explain ~40% of disability of patients with MS (10). However, cervical spinal cord involvement is also associated with marked brain cortical atrophy and in a reduction of the pyramidal and cerebellar tracts, which could further accelerate the neurodegenerative process (11). In fact, some observations provide strong evidence that SC injury, rather than diffuse symmetric injury to the brain's white matter, is the major contributor to motor progression in MS (12).

The status of oligoclonal bands, both OCGB and OCMB, has been related by previous studies with an earlier conversion to SPMS. However, in our multivariate analysis, OCGB were not an independent factor. This is not surprising, as OCGB may be a better biomarker of disease rather than severity, because it is already present in almost 85% of patients with CIS and hence does not discriminate between patients (13).

Regarding OCMB, the prognostic value of OCMB as a risk factor to develop SPMS is somehow expected. Previous works have suggested that somatic hypermutations and class switch in B cells from patients with MS are driven by activation-induced cytidine deaminase (AICD), which is also necessary for the formation of ectopic leptomeningeal follicles (eLF) (14–17). These eLF have been demonstrated in PMS and are linked to cortical atrophy and surrounding demyelination (15, 18). In addition, OCMB have also been related to early predictors such as shorter time to second relapse, early increase in the B-T2L, and early development of brain atrophy, all of which are in turn related to the development of SPMS. Lastly, the importance of the intrathecal synthesis of IgM to the development of sustained disability has been emphasized by several authors, in the form of OCMB, LS-OCMB, or the IgM index. We are aware of the technical limitations to the determination of OCMB, as it requires specific methodology and expertise that might not be available in many clinical settings (8). In this regard, other techniques such as the Link IgM index and free kappa chains in the CSF are being explored to improve the ease of the detection of IgM antibodies in clinical practice. In particular, the IgM index using the Reiber formula has been linked to the number of spinal T2 lesions. However, only 23% of patients showed IgM synthesis with this methodology, compared to 40% of our patients. Reasons for this discrepancy remain to be elucidated but may be due to differences in sensitivity and specificity, the hyperbolic function followed by the Reiber formula (compared to IgM index), or the different spinal cord lesion analysis performed (whole vs. only cervical spinal cord) (9, 17, 19).

Remarkably, the presence of cSC-T2L and positive OCMB results in an absolute risk of SPMS similar to the sum of each one separately. This supports the idea that each variable portrays different and complementary information. Still, patients with OCMB were more likely to have spinal-cord T2 lesions, supporting the idea that intrathecal IgM synthesis is strongly associated with spinal cord involvement and more pronounced neuroaxonal injury in early MS. The independence of this predictor observed in our work may be explained by the longer observation period (19).

In contrast to other studies (20), our final model did not find differences in the risk to develop SPMS in patients starting with “first-line” or he-DMTs. This might be due to the lower number of patients treated with he-DMT (*n* = 15) and because the presence of OCMB might skew treatment toward he-DMT.

We are aware of important limitations that apply to our study, such as the retrospective design, potential bias arising from variable selection, influence from other unadjusted variables (such as influence of relapses, their topography, effect of pregnancy, JC virus titer, progressive multifocal leukoencephalopathy), and the relatively low number of events (i.e., 36 converted to SPMS). On the contrary, the multicenter design, long follow-up, low number of patients lost, and centralization of the MRI and CSF studies give robustness to our results.

In relation to similar studies, our patients had similar characteristics such as percentage fulfilling the 2017 McDonald criteria (53.9%), OCGB positivity (84.6%), OCMB positivity (49%), and distribution of T2 lesions (21), including those within the spinal cord (22). Yet, we had a slightly higher proportion of patients with brain-GEL (56.2%) (23, 24). This warrants further studies to reproduce our findings in other populations, desirably with a larger number of patients or longer follow-up time.

The results of this study cannot be generalized to current cohorts, as standards of treatment have changed substantially in the last 20 years (i.e., patients are treated earlier and more DMT of higher efficacy are available). However, we still think our observations may help to identify patients at a higher risk of evolving to SPMS from baseline.

In conclusion, our work supports previous studies of similar design and observation time, showing that the presence of OCMB and demyelinating lesions within the cervical spinal cord increase the risk for conversion to SPMS and that these variables might be independent of each other. Therefore, we provide class III evidence that performing OCMB determination and cervical cord imaging in patients presenting with CIS might be useful to stratify the risk of SPMS and thus may help in decision-making toward more efficacious treatments.

## Data availability statement

The original contributions presented in the study are included in the article/Supplementary material, further inquiries can be directed to the corresponding author.

## Ethics statement

The studies involving human participants were reviewed and approved by Hospital La Fe of Valencia. The patients/participants provided their written informed consent to participate in this study.

## Author contributions

BC: conception and design of the study, acquisition and analysis of data, and drafting the manuscript and figures. CQ-B, FG, and CA: acquisition and analysis of data, drafting, and critical revision of the manuscript. LL, MC-G, JM, LT, JD, EC, SG-P, LC, RG, JC-V, and FP-M: acquisition and analysis of data. MS: statistical analysis. JC and SC: imaging analysis. All authors contributed to the article and approved the submitted version.

## Funding

This research has been supported by a grant from the Health Institute Carlos III (PI20/01446) and FEDER funding.

## Conflict of interest

The authors declare that the research was conducted in the absence of any commercial or financial relationships that could be construed as a potential conflict of interest.

## Publisher's note

All claims expressed in this article are solely those of the authors and do not necessarily represent those of their affiliated organizations, or those of the publisher, the editors and the reviewers. Any product that may be evaluated in this article, or claim that may be made by its manufacturer, is not guaranteed or endorsed by the publisher.
