# Supplementary material for: Oligoclonal M bands and cervical spinal cord lesions predict early secondary progressive multiple sclerosis
Source: Front Neurol. 2022 Oct 28;13:991596. doi: 10.3389/fneur.2022.991596 (PMC9650197; doi:10.3389/fneur.2022.991596)

**Supplementary material**

Table e-1: Chi-square of the variables explored to determine the degree of dependence.

|  | Age | Sex | ClS | McD.Cr. | Ttc | BT2L | GEL | BST2L | SCT2L | OCGB | OCMB |
| --- | --- | --- | --- | --- | --- | --- | --- | --- | --- | --- | --- |
| Age | 1 | .80 | .02 | .36 | .94 | .85 | .42 | .28 | .63 | .29 | .28 |
| Sex | .80 | 1 | .23 | .17 | .23 | .24 | .24 | .58 | .31 | .10 | .82 |
| CIS | .02 | .23 | 1 | .10 | .54 | .002 | .11 | <.000 | <.000 | .34 | .84 |
| McD.Cr. | .36 | .17 | .10 | 1 | .61 | <.000 | <.000 | .11 | .024 | <.000 | .008 |
| Ttc | .94 | .23 | .54 | .61 | 1 | .39 | .53 | .66 | .73 | .024 | .13 |
| BT2L | ,85 | .24 | .002 | <.000 | .39 | 1 | <.000 | .005 | .083 | <.000 | .006 |
| GEL | .42 | .24 | .11 | <.000 | .53 | <.000 | 1 | <.000 | .043 | <.000 | .007 |
| BST2L | .28 | .58 | <.000 | .11 | .66 | .005 | <.000 | 1 | .92 | .007 | .21 |
| SCT2L | .63 | .31 | <.000 | .024 | .73 | .083 | .043 | .92 | 1 | .049 | .002 |
| OCGB | .29 | .10 | .34 | <.000 | .024 | <.000 | <.000 | .007 | .049 | 1 | <.000 |
| OCMB | .28 | .82 | .84 | .008 | .13 | .006 | .007 | .21 | .002 | <.000 | 1 |

CIS: Clinically isolated syndrome

McD.Cr.: Fulfillment of 2017 McDonald criteria at baseline MRI

Ttc: Treatment initiation as clinically isolated syndrome vs. fullfilling 2017 McDonald criteria

BT2L: brain T2 lesion number stratified in three groups

GEL: presence of gadolinium-enhancing lesions

BST2L: presence of brainstem T2 lesions

SCT2L: presence of cervical spinal cord T2 lesions

OCGB: presence of oligoclonal G bands

OCMG: presence of oligoclonal M bands

Table e-2: Calculation of the log-likelihood beta-coefficient to the models

| Model | Variables in the model | Log-likelihood | Significance |
| --- | --- | --- | --- |
| 1 | Age and CIS | 296.123 | No |
| 2 | Age, CIS, BT2L, BST2L, SCT2L | 287.405 | No |
| 3 | McD..Cri., BT2L, GEL, SCT2L, OCGB, OCMB | **312.471** | SCT2, OCMB |
| 4 | Ttc, OCGB | **331.111** | No |
| 5 | CIS, McD.Cri, BT2L, BST2L, OCGB, OCMB | 261.287 | OCMB |
| 6 | McD.Cri., BT2L, GEL, BST2L, SCT2L, OCGB, OCMB | 309.412 | SCT2, OCMB |
| 7 | CIS, BT2L, GEL,BST2L, OCGB | 281,933 | No |
| 8 | CIS, McD.Cri, Gel, SCT2L, OCGB, OCMB | 261.473 | OCMB |
| 9 | McD.Cri., Ttc, BT2L, GEL, BST2L, SCT2L, OCGB, OCMB | 300.426 | SCT2 |

CIS: Clinically isolated syndrome

McD.Cr.: Fulfillment of 2017 McDonald criteria at baseline MRI

Ttc: Treatment initiation as clinically isolated syndrome vs. fullfilling 2017 McDonald criteria

BT2L: brain T2 lesion number stratified in three groups.

GEL: Presence of gadolinium-enhancing lesions.

BST2L: presence of brainstem T2 lesions.

SCT2L: presence of spinal cord T2 lesions.

OCGB: presence of oligoclonal G bands.

OCMG: presence of oligoclonal M bands.

Table e-3: Omnibus test of model 3 coefficient.

| **Omnibus tests of model coefficients** | | | | |  |  |  |  |  |  |
| --- | --- | --- | --- | --- | --- | --- | --- | --- | --- | --- |
| Log-likelihood -2 | | | | |  |  |  |  |  |  |
| 312,471 | | | | |  |  |  |  |  |  |
| **Omnibus tests of model coefficients^a^** | | | | | | | | | | |
| Log-likelihood -2 | Global | | | Change from previous step | | | | Change from the previous block | | |
|  | Chi-square | df | Sig. | Chi-square | | df | Sig. | Chi-square | df | Sig. |
| **275,847** | 29,072 | 7 | ,000 | 36,624 | | 7 | ,000 | 36,624 | 7 | ,000 |

| **Variables in the equation** | | | | | | | | |
| --- | --- | --- | --- | --- | --- | --- | --- | --- |
|  | B | SE | Wald | df | Sig. | Exp(B) | 95,0% IC for Exp(B) | |
|  |  |  |  |  |  |  | Lower | Higher |
| McD.Cri. | -1,343 | 81,746 | ,000 | 1 | ,987 | ,261 | ,000 | 9,980E+68 |
| BT2L |  |  | 1,268 | 2 | ,531 |  |  |  |
| BT2L(1) | -7,050 | 33,295 | ,045 | 1 | ,832 | ,001 | ,000 | 1,9023E+25 |
| BT2L(2) | -,517 | ,467 | 1,225 | 1 | ,268 | ,596 | ,239 | 1,490 |
| GEL | ,843 | 81,745 | ,000 | 1 | ,992 | 2,324 | ,000 | 8,860E+69 |
| SCT2L | -,903 | ,457 | 3,904 | 1 | ,048 | ,405 | ,166 | ,993 |
| OCGB | -7,126 | 25,592 | ,078 | 1 | ,781 | ,001 | ,000 | 4,88632E+18 |
| OCMB | -1,192 | ,435 | 7,490 | 1 | ,006 | ,304 | ,129 | ,713 |

Table e-4: Omnibus test of model 3 coefficient when OCGB is substituted for age.

| **Omnibus tests of model coefficients** |
| --- |
| Log-likelihood -2 |
| 312,471 |

| **Omnibus tests of model coefficients^a^** | | | | | | | | | |
| --- | --- | --- | --- | --- | --- | --- | --- | --- | --- |
| Log-likelihood -2 | Global | | | Change from previous step | | | Change from the previous block | | |
|  | Chi-square | df | Sig. | Chi-square | df | Sig. | Chi-square | df | Sig. |
| **276,968** | 30,855 | 8 | ,000 | 35,503 | 8 | ,000 | 35,503 | 8 | ,000 |
|  | | | | | | | | | |

| **Variables in the equation** | | | | | | | | |
| --- | --- | --- | --- | --- | --- | --- | --- | --- |
|  | B | SE | Wald | df | Sig. | Exp(B) | 95,0% IC for Exp(B) | |
|  |  |  |  |  |  |  | Lower | Higher |
| McD.Cri. | -,788 | 83,218 | ,000 | 1 | ,992 | ,455 | ,000 | 3,114E+70 |
| BT2L |  |  | 1,504 | 2 | ,471 |  |  |  |
| BT2L (1) | -7,499 | 36,852 | ,041 | 1 | ,839 | ,001 | ,000 | 1,29252E+28 |
| BT2L (2) | -,569 | ,470 | 1,465 | 1 | ,226 | ,566 | ,225 | 1,422 |
| GEL | ,177 | 83,217 | ,000 | 1 | ,998 | 1,194 | ,000 | 8,153E+70 |
| SCT2L | -,931 | ,459 | 4,122 | 1 | ,042 | ,394 | ,160 | ,968 |
| OCMB | -1,481 | ,438 | 11,438 | 1 | ,001 | ,227 | ,096 | ,536 |
| Age_group |  |  | 2,157 | 2 | ,340 |  |  |  |
| Age_group (1) | -,598 | ,482 | 1,538 | 1 | ,215 | ,550 | ,214 | 1,415 |
| Age_group (2) | -,080 | ,476 | ,028 | 1 | ,867 | ,923 | ,363 | 2,346 |

Table e-5. Demographic and clinical characteristics of patients with only OCMB and additional LS-OCMB in the CSF.

|  | **Patients with only OCMB (n=23)** | **Patients with additional LS-OCMB (n=91)** | ***p*-value** |
| --- | --- | --- | --- |
| Age^*^ | 32.0 (7.3%) | 32.6 (9.3) | ns |
| Sex (females) | 19 (82.6%) | 62 (68.1%) | ns |
| Evolution time (years)* | 12.2 (2.4%) | 12.2 (3.0) | ns |
| **McDonald criteria (n, %)** | **13 (59.1**%**)** | **56 (64.4**%**)** | **ns** |
| *CIS syndrome* |  |  |  |
| Myelitis | 10 (43.5%) | 30 (34.9%) |  |
| Optic Neuritis | 6 (26.1%) | 26 (30.2%) |  |
| Brainstem syndrome | 3 (13.0%) | 17 (19.8%) |  |
| Polyregional syndrome | 2 (8.7%) | 5 (5.8%) |  |
| Hemispheric syndrome | 2 (8.7%) | 8 (9.3%) |  |
| *MRI biomarkers* |  |  |  |
| Total number of T2L* | 13.7 (14.1) | 16.0 (13.7) | ns |
| NT2L (grouped) |  |  | .016 |
| 0-1 B-T2L | 3 (13.6%) | 1 (1.1%) |  |
| Between 2-9 B-T2L | 8 (36.4%) | 30 (34.5%) |  |
| > 9 B-T2L | 11 (50.0%) | 56 (64.4%) |  |
| Scans with brain GEL | 15 (68.2%) | 56 (64.4%) | ns |
| Scans with BS-T2L | 10 (43.5%) | 44 (51.8%) | ns |
| Scans with SCT2L | 16 (72.7%) | 56 (67.5%) | ns |
| Number of SCT2L* | 1.0 (1.0) | 1.4 (1.4) | ns |
| *Treatment* |  |  |  |
| Treated patients | 21 (91.3%) | 91 (100%) | ns |
| Treated as CIS | 11 (52.4%) | 48 (54.5%) | ns |
| *Actual treatment* |  |  | ns |
| No treatment | 2 (8.7%) | 1 (1.1%) |  |
| 1^sr^ line therapies | 8 (34.8%) | 45 (48.4%) |  |
| 2^nd^ line therapies | 13 (56.5%) | 46 (50.5%) |  |
| EDSS* at last observation | 2.5 (1.9) | 2.6 (1.9) | ns |
| Phenotype at last observation |  |  | ns |
| CIS | 1 (4.3%) | 0 |  |
| RRMS | 17 (73.9%) | 73 (80.2%) |  |
| SPMS | 5 (21.7%) | 18 (19.8%) |  |

*Mean and standard deviation.

OCMB: Oligoclonal M bands; LS-OCMB: Lipid-specific oligoclonal M bands; B-T2L: brain T2 lesions; NT2L: number of brain T2 lesions; GEL: gadolinium enhancing lesions in the brain. BS: brainstem or cerebellar T2 lesions; SC-T2L: spinal cord T2 lesions; CIS: clinically isolated syndrome; RRMS: relapsing-remitting multiple sclerosis; SPMS: secondary progressive multiple sclerosis.

Table e-6: Multivariate Cox regression analysis of time to reach a diagnosis of secondary progressive multiple sclerosis (SPMS), adjusted by age, gender (female vs. male), presence of spinal cord T2 lesions (SC-T2L), oligoclonal M bands in CSF (OCMB) and baseline EDSS.

|  | | | | | | | | |
| --- | --- | --- | --- | --- | --- | --- | --- | --- |
|  | B | ET | Wald | gl | Sig. | HR | 95.0% CI | |
|  |  |  |  |  |  |  | lower | higher |
| Gender | -.136 | .425 | .103 | 1 | .749 | 1.146 | .498 | 2.635 |
| Age | .024 | .020 | 1.484 | 1 | .223 | 1.024 | .985 | 1.065 |
| SC-T2L | 1.111 | .473 | 5.503 | 1 | .019 | 3.037 | 1.201 | 7.681 |
| OCMB | 1.349 | .452 | 8.889 | 1 | .003 | 3.853 | 1.587 | 9.354 |
| Baseline EDSS | 1.068 | .182 | 34.601 | 1 | .000 | 2.910 | 2.039 | 4.155 |

Table e-7: Kaplan-Meier analysis of time to SPMS according to no treatment at any time, starting with first-line DMT or with he-DMT.

|  | n | Events | Censored | | No treatment | | First-line DMT | |
| --- | --- | --- | --- | --- | --- | --- | --- | --- |
|  |  |  | N | % | Chi-square | p value | Chi-square | p value |
| No treatment | 36 | 0 | 36 | 100.0% | - | - | - | - |
| First-line DMT | 211 | 31 | 180 | 85.3% | 4.511 | .034 | - | - |
| He-DMT | 15 | 5 | 10 | 66.7% | 12.181 | .000 | 7.867 | .005 |
| Global | 262 | 36 | 226 | 86.3% | - | - | - | - |

Table e-8: Multivariate Cox regression analysis of time to reach SPMS according to initial treatment (first-line DMT vs. he-DMT).

|  | B | ET | Wald | df | p value | HR | 95% CI | |
| --- | --- | --- | --- | --- | --- | --- | --- | --- |
|  |  |  |  |  |  |  | Lower | Higher |
| SCT2L | .874 | .457 | 3.651 | 1 | .056 | 2.395 | .978 | 5.868 |
| Treatment with he-DMT | 1.278 | .501 | 6.511 | 1 | .011 | 3.591 | 1.345 | 9.587 |
| OCMB | 1.297 | .435 | 8.877 | 1 | .003 | 3.659 | 1.559 | 8.589 |

|  | **Patients whose clinical syndrome could not be determined (n=13)** | **Patients with a determined clinical syndrome (n=249)** | **p*-value |
| --- | --- | --- | --- |
| Age (mean, SD) | 41.6 (9.9) | 31.9 (8.8) | 0.004 |
| Sex (% females) | 92.3 | 69.5 | ns |
| McDonald 2017 (%) | 61.5 | 53.5 | ns |
| Basline EDSS (mean, SD) | 1.6 (.9) | 1.5 (.9) | ns |
| EDSS at year 5 (mean, SD) | 1.4 (.6) | 1.9 (1.1) | ns |
| EDSS at year 10 (mean, SD) | 2.4 (1.5) | 1.9 (1.4) | ns |
| OCGB (% positive) | 100 | 83.7 | ns |
| OCMB (% positive) | 53.8 | 48.8 | ns |
| T2LN (mean, SD) | 10.9 (3.6) | 10.7 (6.6) | ns |
| SCT2L (%, with T2L) | 30.8 | 56.6 | ns |
| Time to SPMS in years (mean, SD) | 9.0 (4.3) | 10.9 (3.6) | ns |

Supplementary e-9: Demographic and clinical characteristics of patients in which non-detemined vs. determined clinical syndrome.

*p-value of student t or chi-square tests performed on quantitative and dichotomic variables, respectively.

OCGB: oligoclonal G bands; OCMB: Oligoclonal M bands; T2LN: number of brain T2 lesions; SCT2L: Presence of spinal cord T2 lesions.

Figure e-1: Graphic representation of the Univariate Cox regression analysis of the potentially informative variables.


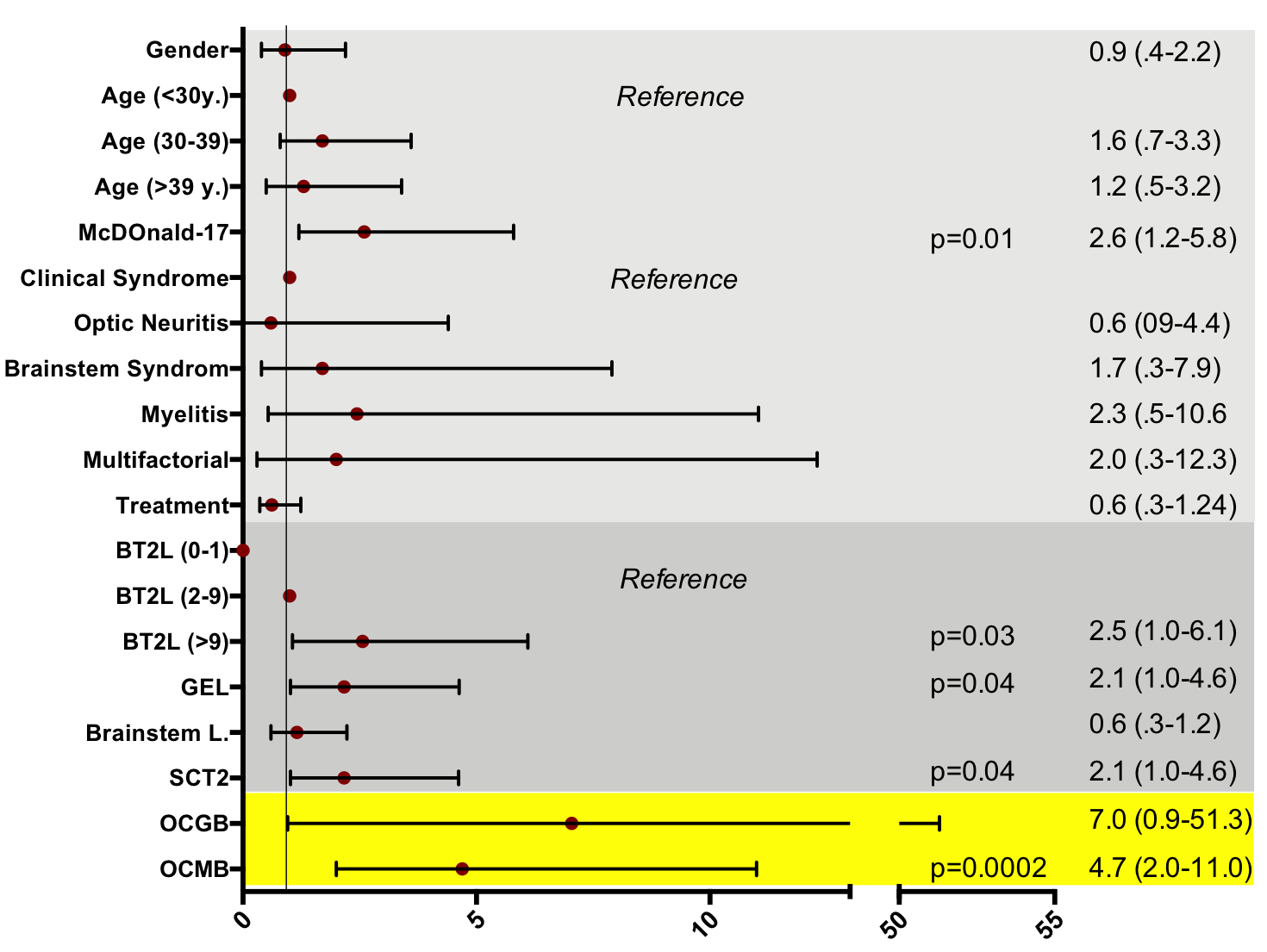

Supplement: Supplementary file 1 [file Data_Sheet_1.docx]
